# Corticosteroid induced mania with psychotic symptoms

**DOI:** 10.1192/j.eurpsy.2021.2002

**Published:** 2021-08-13

**Authors:** F. Gomes Tavares, M. Viseu De Carvalho, M. Pinto, C. Solana, S. Batista

**Affiliations:** Departamento De Psiquiatria E Saúde Mental - Faro, Centro Hospitalar Universitário do Algarve, Faro, Portugal

**Keywords:** corticosteroid, manía, psychosis, prevention

## Abstract

**Introduction:**

Corticosteroids may induce psychiatric symptoms (agitation, fear, hypomania, insomnia, irritability, labile mood, pressured speech and restlessness) with incidence rates ranging from 1,8% to 57%. We present a case of corticosteroid-induced mania and psychosis.

**Objectives:**

Non-systematic review on corticosteroid therapy induced psychiatric symptoms. Analysis and comparison of a patient’s case with the existing literature.

**Methods:**

Case report and a non-systematic review through databases as Pubmed, UpToDate, Medscape, between 2000 and 2020.

**Results:**

We present a female 70 year-old patient without psychiatric background, diagnosed with Rhizomelic Pseudopolyarthritis, who started treatment with prednisone 20 mg. During the third month of treatment the patient started progressively worse behavior changes (such as destruction of the neighbor’s property), developed persecutory delusions, decreased sleep and increased energy. The patient was committed to our psychiatric ward and started on diazepam 10 mg and olanzapine 15 mg per day. Despite introduction of antipsychotics, which has evidence for mood stabilization, the patient maintained the symptoms, so it was necessary to gradually reduce corticosteroids until symptomatic control.

**Conclusions:**

Psychosis (24%), hypomania and mania (35%), are the most common psychiatric reactions to corticosteroid therapy. Several studies show that even a low dosage may induce psychiatric disturbances, most frequently during the first two weeks of treatment. However, as reported in this case, symptoms may occur at any time. Thus, a multidisciplinary team, as well as training of professionals from different specialties, such as psychiatry, rheumatology and endocrinology, are needed, since these syndromes may be confused with pure psychiatric conditions and consequently delay treatment and compromise prognosis.

**Disclosure:**

No significant relationships.

